# Stump Ectopic Pregnancy: A Rare Presentation

**DOI:** 10.7759/cureus.60859

**Published:** 2024-05-22

**Authors:** Anju Singh, Abhay Kumar, Rimpi Singla, Rashmi Bagga, Parikshaa Gupta

**Affiliations:** 1 Obstetrics and Gynecology, Postgraduate Institute of Medical Education and Research, Chandigarh, IND; 2 Cytopathology and Gynecologic Pathology, Postgraduate Institute of Medical Education and Research, Chandigarh, IND

**Keywords:** post-salpingectomy, ruptured ectopic pregnancy, laparotomy, tubal stump ectopic, ectopic pregnancy

## Abstract

Ectopic pregnancy is a significant cause of maternal morbidity and mortality in women of reproductive age group. Tubal ectopic in an unstable patient is a medical emergency. Tubal stump ectopic is a rare presentation. It is difficult to diagnose. Early diagnosis can prevent significant morbidity and mortality. Here, we present a case of ruptured tubal stump ectopic pregnancy in a 33-year-old female who had undergone salpingectomy previously for ectopic pregnancy.

## Introduction

Ectopic pregnancy is defined as a pregnancy that occurs outside the uterine cavity. It accounts for approximately 2% of all reported cases [1]. The most common location of ectopic pregnancy is the fallopian tube, which accounts for almost 90% [2]. It usually presents as hemodynamic instability, vaginal bleeding, or acute abdomen. Ectopic pregnancy on the residual stump has been reported sporadically [3,4]. Stump ectopic pregnancies are very rare, and account for 0.4% of all ectopic pregnancies [5]. Ultrasonography is effective for the diagnosis of stump ectopic. Till now, in the literature, only a handful of cases have been described. Management options include beta HCG monitoring, laparoscopy, or laparotomy. Here we present the successful management of a case of ruptured tubal stump ectopic who had undergone salpingectomy previously for ectopic pregnancy.

## Case presentation

**Figure 1:**
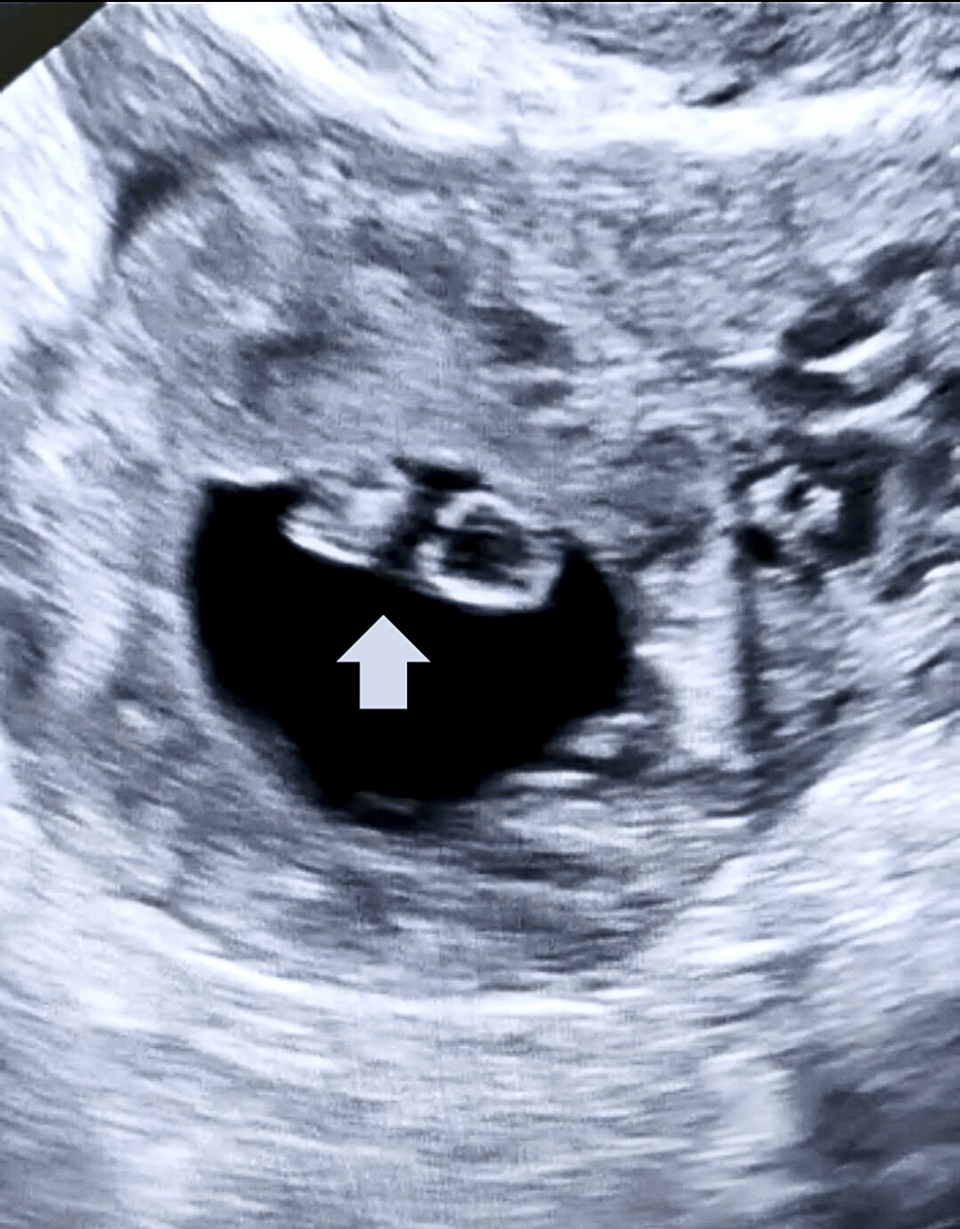
Tubal stump ectopic with a fetal pole (arrow)

A 33-year-old female gravida 4, para 1, presented to our emergency department with an ultrasound report of right ectopic. She was married for seven years, and this was her spontaneous conception. She went for a routine antenatal ultrasound examination and was diagnosed with a right ectopic pregnancy. She had no history of pain in the abdomen, bleeding per vaginum, dizziness, or syncope. In her obstetrical history, she had one cesarean five years ago and a laparotomy for a ruptured ectopic two years ago. Her documentation of the previous ectopic showed a right salpingectomy. On examination, she had a BP of 110/70 mm hg, PR of 96/min, and RR of 16/min. Respiratory and cardiovascular examinations were normal. On abdomen examination, the abdomen was soft and non-tender. Using a speculum, the cervix and vagina were found to be grossly normal, and no bleeding was noted. On bimanual examination, the uterus was retroverted, normal in size, the cervix was in mid-position, cervical motion tenderness was present, and right forniceal tenderness was present. Ultrasound showed a right adnexal mass of 2.8x2.6 cm with fetal pole corresponding to 6±3 weeks (Figure 1), with free fluid present in the pelvis.

**Figure 2:**
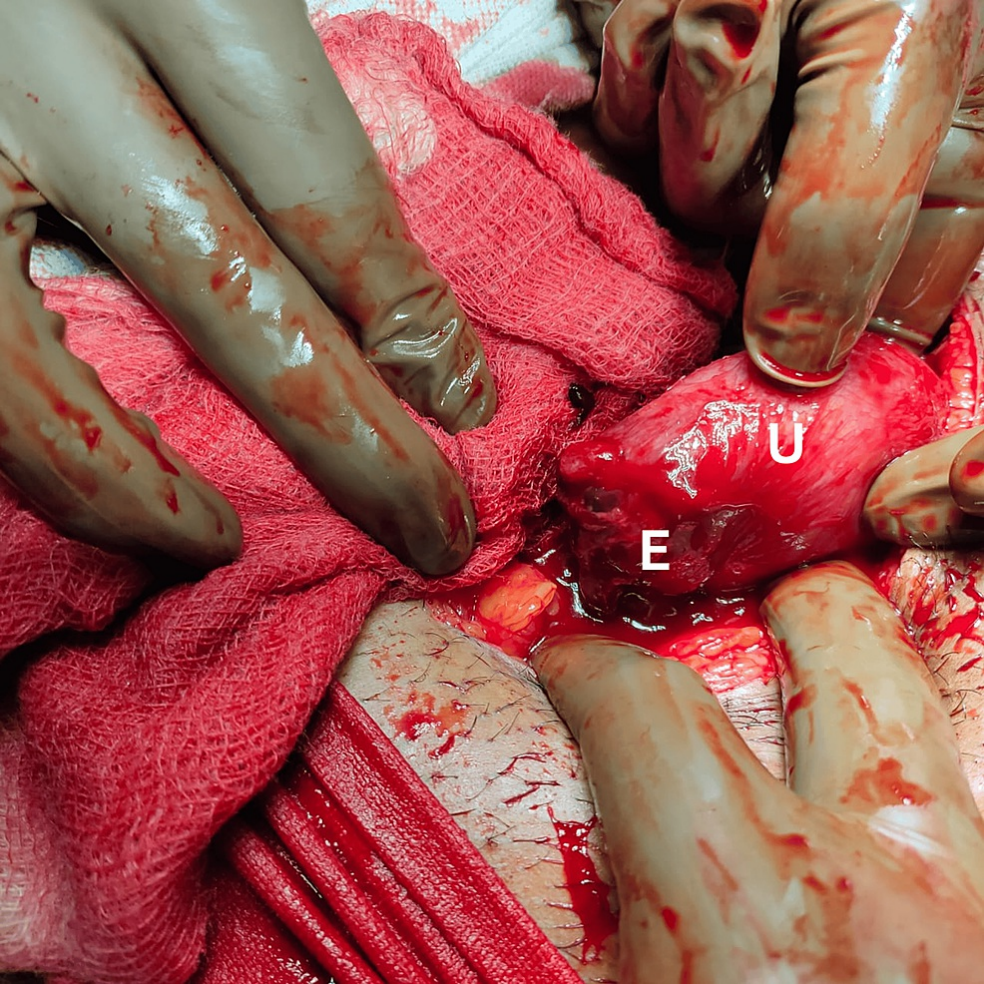
Ruptured stump ectopic U, uterus; E, bleeding stump ectopic

Her hemoglobin was 10.6 gm/dL with platelets of 2.1 lac. Her coagulation, renal, and liver functions were normal. Based on her high hCG (3500) and the presence of free fluid, the decision of laparotomy was taken. An emergency laparotomy was performed. Intraoperative findings showed a right stump ectopic of 3x3 cm with bleeding present from it (Figure 2).

**Figure 3:**
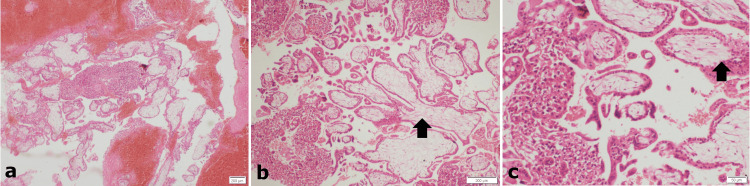
Section from the fallopian tubal stump showing large areas of hemorrhage, multiple first-trimester chorionic villi (arrow), and islands of trophoblastic tissue

The stump was cut and ligated, hemostasis was secured, 300 cc of blood was present, left tube and ovary were grossly normal. The patient withstood the procedure well. The postoperative period was uneventful. The patient was discharged on day 3. Histopathology consistent with the stump ectopic (Figure 3) and hCG levels came down on subsequent visits. 

## Discussion

An ectopic pregnancy is an extrauterine pregnancy. It carries a high risk of morbidity and mortality when not recognized and treated early. The ampullary area is the most often seen location for ectopic pregnancies, accounting for 92% of cases. The interstitium, cornua, cervix, ovaries, and peritoneum are the other, less frequent locations [6]. History of the previous ectopic pregnancy increases the risk of recurrence. The risk of recurrence increases up to 25% in two or more ectopic pregnancies [7]. It usually presents as amenorrhea with pain in the abdomen or vaginal bleeding. The index case had a history of previous ruptured ectopic for which a salpingectomy was performed. She presented early with no symptoms and was aware of the recurrence of ectopic, so she got ultrasonography early, which revealed ectopic on the same side. Several theories have been proposed to explain the pathophysiology of stump ectopic pregnancy. According to the external chemotactic theory, implantation at the tubal stump happens when a fertilized ovum passes through a foramen that forms at the stump [5]. An alternative hypothesis suggests that the fertilized ovum from the contralateral normal fallopian tube causes the implantation [8]. Ultrasound examination is an effective tool for diagnosing, but it poses difficulty in diagnosis as the ovary is nearly situated and ovarian follicles can be confused as stump ectopic. The gold standard of management is surgery, whether laparoscopy or laparotomy, and excision of the stump, as done in our case. The management options can also include conservative management and methotrexate injection. According to Lau and Tulandi, methotrexate therapy had an 83% success rate while surgical treatment had a 100% overall success rate [9]. Certain recommendations aim to reduce the chance of ectopic pregnancy recurrence in a residual tube following tubectomy. These include minimizing the remnant tube's length and achieving an adequate distance closer to the tip of the remnant tube using diathermy or the use of clips.

## Conclusions

Tubal stump ectopic is a rare presentation. It poses difficulty in diagnosis and management. Even after the salpingectomy, patients are at risk of developing recurrent ectopic. Therefore, the possibility of stump ectopic should be kept in mind while doing the ultrasound. While performing the salpingectomy, the length of the remanent tube should be minimized.
